# Healthy Properties of a New Formulation of Pomegranate-Peel Extract in Mice Suffering from Experimental Autoimmune Encephalomyelitis

**DOI:** 10.3390/molecules27030914

**Published:** 2022-01-28

**Authors:** Giulia Vallarino, Annalisa Salis, Elena Lucarini, Federica Turrini, Guendalina Olivero, Alessandra Roggeri, Gianluca Damonte, Raffaella Boggia, Lorenzo Di Cesare Mannelli, Carla Ghelardini, Anna Pittaluga

**Affiliations:** 1 Department of Pharmacy, University of Genoa, Viale Cembrano, 4 I, 16148 Genoa, Italy; vallarino@difar.unige.it (G.V.); turrini@difar.unige.it (F.T.); olivero@difar.unige.it (G.O.); roggeri@difar.unige.it (A.R.); pittalug@difar.unige.it (A.P.); 2 Department of Experimental Medicine, Section of Biochemistry, University of Genova, Viale Benedetto XV 1, 16132 Genoa, Italy; annalisa.salis@unige.it (A.S.); Gianluca.Damonte@unige.it (G.D.); 3 Department of Neuroscience, Psychology, Drug Research and Child Health, Neurofarba, Pharmacology and Toxicology Section, University of Florence, Viale Pieraccini 6, 50139 Florence, Italy; elena.lucarini@unifi.it (E.L.); lorenzo.mannelli@unifi.it (L.D.C.M.); carla.ghelardini@unifi.it (C.G.); 4 Center of Excellence for Biomedical Research (CEBR), University of Genova, Viale Benedetto XV 9, 16132 Genoa, Italy

**Keywords:** pomegranate peels, ellagic acid, multiple sclerosis, inflammation, demyelination, astrocytosis

## Abstract

A new formulation of a pomegranate-peel extract (PEm) obtained by PUAE (Pulsed Ultrasound-Assisted Extraction) and titrated in both ellagic acid (EA) and punicalagin is proposed, characterized and then analyzed for potential health properties in mice suffering from the experimental autoimmune encephalomyelitis (EAE). PEm effects were compared to those elicited by a formulation containing EA (EAm). Control and EAE mice were chronically administered EAm and Pem dissolved in the drinking water, starting from the day 10 post-immunization (d.p.i.), with a “therapeutic” protocol to deliver daily 50 mg/kg of EA. Treated EAE mice did not limit their daily access to the beverage, nor did they show changes in body weight, but they displayed a significant amelioration of “in vivo” clinical symptoms. “Ex vivo” histochemical analysis showed that spinal-cord demyelination and inflammation in PEm and EAm-treated EAE mice at 23 ± 1 d.p.i. were comparable to those in the untreated EAE animals, while microglia activation (measured as Ionized Calcium Binding Adaptor 1, Iba1 staining) and astrocytosis (quantified as glial fibrillar acid protein, GFAP immunopositivity) significantly recovered, particularly in the gray matter. EAm and PEm displayed comparable efficiencies in controlling the spinal pathological cellular hallmarks in EAE mice, and this would support their delivery as dietary supplementation in patients suffering from multiple sclerosis (MS).

## 1. Introduction

Special dietary regimes and/or supplementations are attracting more and more the attention of researchers to support therapy and to improve patients’ life quality. In particular, the use of diets and dietary supplements has largely increased in recent years in patients suffering from multiple sclerosis (MS), despite the recent analysis from Farinotti et al. that led to the conclusion that dietary supplementation with polyunsaturated fatty acids, antioxidants and vitamins did not exert clear beneficial effects on the survival in MS patients [1].

Among foods considered rich in bioactive compounds that could be beneficial to the course of MS, there is the pomegranate, which recently gained huge popularity as a nutraceutical source, becoming a high-value crop [2]. Promising results have been reported not only in the treatment of cardiovascular disease, diabetes, obesity, prostate cancer and intestinal inflammatory disease, but also in central neuroinflammation and in neurodegenerative processes [3,4,5,6], including those related to MS [7,8,9].

The potential health-promoting properties of pomegranate fruit were reviewed by Aktar et al. (2015) [10], who emphasized that the nutraceutical properties are not restricted to its edible portion but are shared by different parts of the fruit (i.e., peels and seeds) and even of the tree (i.e., buds, barks and leaves). In fact, fruit waste and by-products (i.e., bagasse, peels, stems, shells and seeds), which can represent more than 50% of the weight of fresh fruit, as for pomegranate, often have a higher bioactives content than the final commercial product [11]. Particularly, both the pomegranate exocarp and mesocarp are particularly rich in ellagitannins (ETs), which are hydrolyzable tannins containing ellagic acid (EA) as common aglycone [12]. ETs are supposed to act through different pathways in the prevention and treatment of several diseases, and, among them, a and b punicalagins are peculiar to the pomegranate phytocomplex, and they are by far more abundant in peels than in arils, where they account for more than 50% of their antioxidant activity [13]. The pomegranate ETs include also punicalin, pedunculagin, gallagic acid and other minor hydrolysable tannins, sharing the properties of being hydrolyzed “in vivo” to EA and further metabolized from gut microbiota to urolithins (mainly A and B urolithins), which can be absorbed, thus reaching several biological targets where the effect of pomegranate’s tannins appears [14,15]. Both ETs and EA are usually considered as nutraceuticals because their dietary intake leads to relevant biological effects both in animal models of pathology and in humans. 

The use of EA preparations is, however, questioned because of its poor aqueous solubility that would correspond to a low oral bioavailability [16]. Boggia and colleagues recently proposed a new approach to increase the EA aqueous solubility entrapping it in a food-compatible matrix and realizing an EA solid microdispersion (EAm) that can be orally administered to mice dissolved in the drinking water [17]. Very recently, the authors demonstrated that the chronic administration of EAm significantly recovered synaptic and inflammatory signs that develop in the CNS of aged rodents [18]. 

In this research, a new pomegranate-peel extract (namely PEm), whose phytocomplex was chromatographically elucidated, was realized by combining a direct sonication process (PUAE: Pulsed Ultrasound-Assisted Extraction) of pomegranate peels, in only water as extraction solvent, and a formulative step by spray-drying with a pectin’s enrichment both to stabilize the formulation and to mask the very astringent taste due to the high content of tannins. According to the twelve principles of green chemistry established by the United States Environmental Protection Agency [19] and the six principles of green extraction [20], ultrasounds through the cavitation process allow us to extract, in a very short time, with high reproducibility and with greater extraction yield compared to the traditional extraction methods, many bioactive compounds from different plant and food products [21,22]. Particularly, PUAE has been recently proved to be an efficient and sustainable technique to extract the polyphenolic fraction (i.e., ETS) from pomegranate by-products (external skins and internal marc) [16,17].

In 2018, Savikin et al. [23] fractionated the pomegranate extracts by a liquid/liquid partition and isolated fractions with different biological activities. Besides the polyphenolic fraction (rich in EA and ETs), the polysaccharide fraction was also found to be biologically active, being involved in the immunostimulatory effects elicited by the phytocomplex [24]. For this reason, in this research, we proposed a new formulation combining ETS and EA from pomegranate peel with pectin as an example of polysaccharides belonging to soluble fiber, which can exert healthy protective effects, including the promotion of the ETs metabolism to urolithins [25]. In addition to the emerging use as a functional health-promoting ingredient, pectin is traditionally widely used both in food productions and in food ingredient formulations, since it is a safe food additive (E440) that is endowed with gelling and thickening properties, with no limit on acceptable daily intake [26]. Moreover, similar to pomegranate peels, pectin represents an agro-industrial waste (i.e., citrus fruits and apple pomaces) obtained from several fruit-processing applications [27]. 

Experimental autoimmune exncephalomyelitis (EAE) is an animal model of MS widely used in preclinical studies to assess the efficacy of therapeutic approaches for this disease. The model recapitulates, particularly at the spinal cord level, most of the histopathological signs of inflammation and demyelination observed in patients suffering from the non-remitting form of MS [28,29]. 

EA, as well as pomegranate extracts, came to the interest of neuroscientists because of their anti-inflammatory and antioxidants properties. In 2015, Binyamin and colleagues provided evidence that a nanodroplet formulation of pomegranate seeds oil (the Nano-PSO, titred for the EA content) administered via gavage reduces the clinical signs in the EAE mice, also ameliorating spinal lipid oxidation and inflammatory infiltrates at the acute stage of the disorder [30]. Two years later, a pomegranate-peel extract obtained with aqueous-ethanol extraction was administered intraperitoneally to EAE rats, starting from the asymptomatic stage of the disease (8 days post-immunization, d.p.i.). The extract ameliorated the clinical score, reduced the release of the cytokine IL17 in draining CD4^+^ T cells purified from the popliteal lympho-node and diminished spinal-cord inflammatory infiltrates [31]. Finally, very recently, Kiasalari and colleagues (2021) [32] administered orally (via gavage) EAE mice with EA (50–10 mg/kg/day) emulsified in Cremophor. The authors observed a positive trend in “in vivo” (i.e., loss of weight and clinical score) and in “in vitro” (cytokine production and histopathological staining) parameters in the treated EAE mice. The beneficial effect of EA was also confirmed in the cuprizone-treated mice, which is a model of demyelinating disorder [32,33]. In a whole, these results were promising but typified by a huge variability depending on the formulation, on the timing of the treatment and on the route of administration that in some cases was invasive (gavage, i.p.) and could reverberate negatively on the course of the disease.

The PEm formulation dissolved in the drinking water was chronically orally administered to EAE mice, and its activity was compared to that of the single aglycone’s formulation (EAm) previously described by the authors of Reference [18].

## 2. Results

### 2.1. Chemical and Chromatographic Characterization of Pem

The EA formulation (EAm) previously described by the authors of Reference [18] represented a “trivial model” to administer the phytocomplex present in the pomegranate fruit peels extracts. In fact, EA is the common aglycone present within the structures of all the ETs in the phytocomplex, and the low-methoxyl pectin used to produce EAm mimic this polysaccharide fraction. In this research, the whole pomegranate-peel phytocomplex was formulated by realizing a solid microdispersion by spray-drying, whose preliminary characterization was reported in Table 1.

The aqueous extract from pomegranate peels lacks long-term stability; therefore, it has been formulated in order to stabilize it over the time. Spray-drying, widely used in the food industry to convert a liquid state into a powder product, due to its efficiency and low cost, has been chosen as a microdispersion technique. This technology allows bioactive phenolic compounds extracted from pomegranate peels to be physically wrapped in a protective “wall material” of pectin to protect them from nutritional deterioration and increase the shelf life of the product. Pectin is widely used in food productions and in food formulations, and it is recommended by the FAO/WHO committee on food additives as a safe additive with no limit on acceptable daily intake [34]. Furthermore, pectin can also be extracted from agro-industrial waste, such as citrus waste, but also pomegranate peels can be a good source for pectin extraction, in view of a whole circular-economy strategy [35]. 

The powder obtained with the microdispersion process was stable (moisture content less than 15%), and it maintained a good solubility in water, as reported in Table 1. In addition, the present formulation preserved its antiradical activity, which was checked monthly during a period of 6 months of storage at room temperature and away from light. Particularly, the radical scavenging activity (RSA), expressed as mean of ascorbic acid equivalent + standard deviation, is also reported in Table 1.

Moreover, PE and the corresponding PEm were analyzed by using HPLC–DAD to evaluate their content (expressed as μg/mL) in EA, the common aglycone to all ETs of the phytocomplex, and punicalagin, as marker compound to evaluate the total amount of pomegranate ETs (Table 2).

As reported in Table 2, the formulation process by spray-drying guaranteed an encapsulation efficiency (EE%) of the pomegranate phytocomplex equal to about 15%.

**Figure 1:**
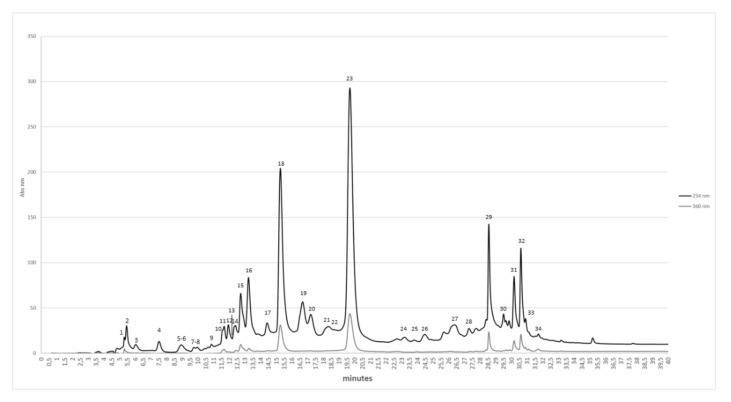
HPLC chromatograms identified by ESI/MS–MS/MS analysis, at 254 nm (black line) and 360 nm (gray line). The numbering corresponds to that reported in Table 3.

Moreover, the HPLC sample profiling (Figure 1), characterized by full scan and tandem electrospray mass spectrometry (ESI/MS–MS/MS), allowed us to identify thirty-four compounds, mainly polyphenols.

The molecules were identified according to their fragmentation pattern that, besides other parameters, was reported for each molecule in Table 3. The following table shows a large presence of ETs and flavonoids, as reported in various phytochemical studies carried out of different parts of the pomegranate [36,37,38].

### 2.2. Pharmacological Activity of PEm and EAm in EAE Mice: “In Vivo” Effects

**Figure 2:**
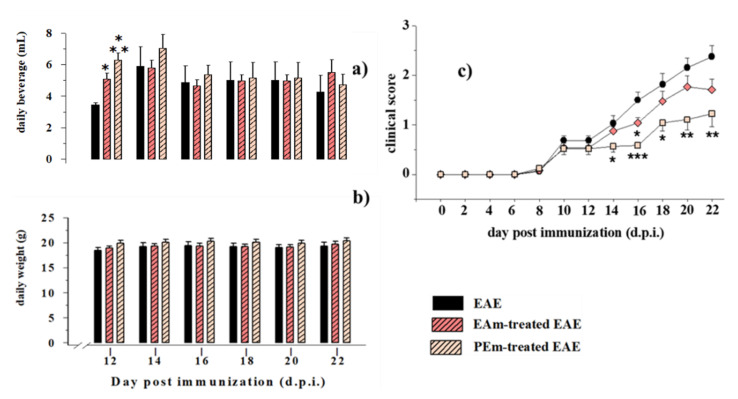
Effects of “in vivo” EAm and PEm treatments on daily beverage intake, weight and clinical score of EAE mice. (**a**) Daily beverage intake (mL) in untreated (*n* = 8 mice), EAm-treated (*n* = 12 mice) and PEm-treated (*n* = 12) EAE mice. The daily intake is expressed as mean ± SEM of the drinking solution (mL) taken up. (**b**) Animal weight (g) in untreated, EAm-treated and PEm-treated EAE mice (number of mice for each group as above). The values are expressed as mean ± SEM of the animal weight (g). (**c**) Clinical scores of untreated, EAm-treated and PEm-treated EAE mice. PEm and EAm treatment started at 10 d.p.i. (see arrow). The clinical score is evaluated as indicated in the Method section, and it is expressed as mean ± SEM (number of mice for group as above). All of these values were measured every two days, at the indicated day, post-immunization (d.p.i.). Note: * *p* < 0.05 vs. untreated EAE mice, ** *p* < 0.01 vs. untreated EAE mice and *** *p* < 0.001 vs. untreated EAE mice.

Based on the recent beneficial effects observed by orally administering young and old mice with the EAm formulation dissolved in the drinking water [18], we adopted this route of administration to evaluate the impact of PEm and EAm in EAE mice. The approach is particularly appropriate, since it permits the drug treatment, avoiding any constriction and/or stressing manipulation that could interfere with the progression of the disease, i.e., by reducing the mice resilience to illness. Experiments were dedicated to investigate whether and to what extent the new PEm formulation (formulated to deliver 50 mg/kg/day of EA) and the EAm (50 mg/kg/day) dissolved in the drinking water could be beneficial in EAE mice. As already observed in aged mice [18], the EAE animals did not limit their daily access to the beverage; rather, the amount of the drunk EAm and PEm solutions increased within the first two days of treatment and then rapidly recovered to the volumes of water taken up daily by the untreated EAE mice (Figure 2a). The EAm and the PEm treatments failed to significantly affect the weight of the EAE mice (Figure 2b), but they caused a constant amelioration of the gravity of the clinical score that was particularly relevant in the PEm-treated EAE mice and, to a lesser extent, in the EAm-treated ones (Figure 2c). Both EAm and PEm treatments failed to significantly affect the drunk volume, as well as the weight of the non-immunized control mice (not shown).

### 2.3. Pharmacological Activity of PEm Compared to EAm in EAE Mice: “Ex Vivo” Assessments

Encouraged by the promising “in vivo” observations, “ex vivo” experiments were carried out to quantify the impact of EAm and PEm administration on markers of demyelination and inflammation, as well as on reactive astrocytosis/gliosis, lymphocyte infiltration and microglia activation.

**Figure 3:**
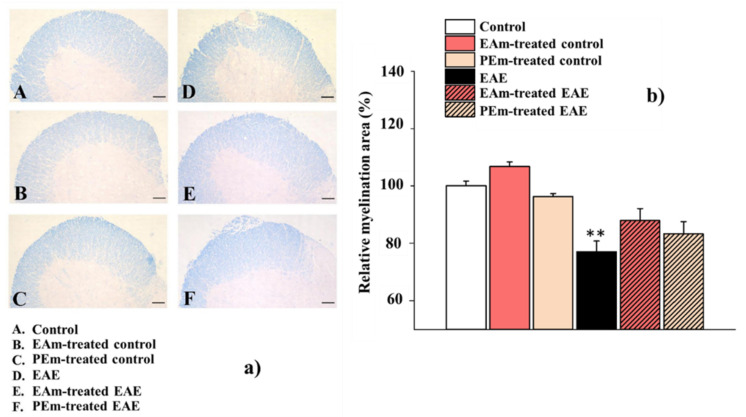
Effects of “in vivo” EAm and PEm treatments on the demyelination in the spinal cord of EAE mice. (**a**) Representative images of ventral-horn white matter marked by Luxol Fast Blue staining (total magnification, 100×; scale bar, 100 µm). (**b**) Quantitative evaluation of average LFB burden. Results are expressed as mean ± SEM of 4 animals per group and controls arbitrary taken as 100%. Note: ** *p* < 0.01 vs. control.

First, we analyzed the demyelination in the lumbar spinal cord. We focused on this region since the EAE-related immunohistopathological signs are particularly evident at this level [39]. Demyelination was quantified with Luxol Fast Blue (LFB) staining in spinal-cord tissue sections, which showed a remarkable demyelination in the EAE group at the acute stage of disease (23 d.p.i) when compared to controls (EAE mice, 77.00 ± 3.73 % vs. control group taken as 100%). Both EAm and PEm treatments partially, but not significantly, reduced the damage to the myelin sheath (EAm treated EAE mice, 87.90 ± 4.19 %; PEm-treated EAE mice, 83.30 ± 4.21 %; Figure 3a,b).

**Figure 4:**
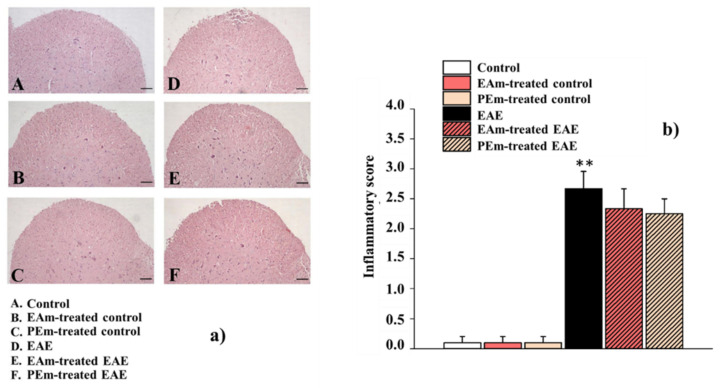
Effects of “in vivo” EAm and PEm treatments on inflammation in the spinal cord of EAE mice. (**a**) Representative images of ventral spinal cord marked by Hematoxylin and Eosin staining (total magnification: 100×. Scale bar: 100 µm) and quantitative evaluation of inflammatory infiltrate. The values represent the mean ± SEM of 4 animals per group. (**b**) Quantitative evaluation of inflammatory infiltrate. The values represent the mean ± SEM of 4 animals per group. Note: ** *p* < 0.01 vs. control.

Next, Hematoxylin and Eosin (H&E) staining was performed to evaluate the inflammatory infiltration, another typical hallmark of the EAE model. No differences were observed among all the EAE groups, suggesting that the inflammatory process persists following the treatments (EAm-treated (2.33 ± 0.33) and PEm-treated (2.25 ± 0.25) EAE mice vs. untreated EAE mice (2.67 ± 0.29); Figure 4a,b).

**Figure 5:**
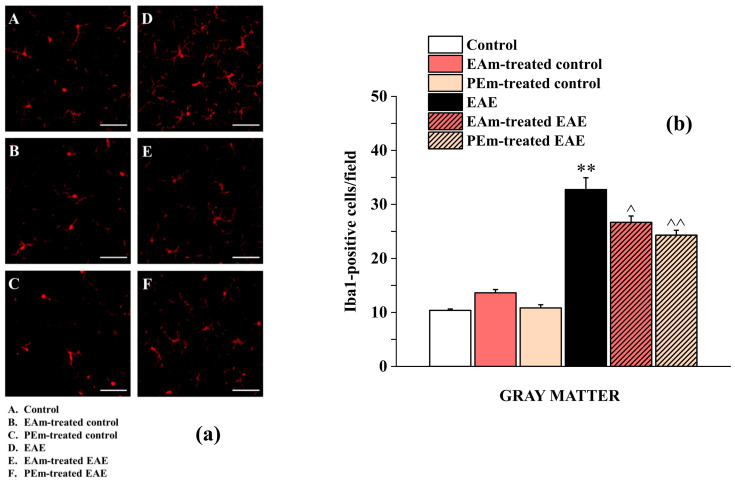
Effects of “in vivo” EAm and PEm treatments on the microglial profile in the spinal cord. (**a**) Representative images obtained by Iba-1 immunofluorescence histochemistry of the ventral spinal cord, lumbar portion (total magnification, 400×; scale bar, 50 µm). (**b**) Quantitative analysis for Iba-1-positive cells/field in the ventral gray matter. Results are expressed as mean ± SEM of *n* = 4 animals per group. Note: ** *p* < 0.01 vs. control group; ^ *p* < 0.05 or ^^ *p* < 0.01 vs. EAE group.

To evaluate the functional status of spinal microglia, Ionized Calcium Binding Adaptor 1 (Iba-1) marker was probed. As shown in Figure 5a,b, the EAE group exhibited a significant increased number of Iba-1 positive cells (33 ± 2) in comparison to the control group (control, 10 ± 1; EAm-treated control, 14 ± 1; PEm-treated control, 11 ± 1). EAm (27 ± 1) and PEm (24 ± 1) treatments prevented the microglial response to the CNS autoimmune inflammatory disease.

**Figure 6:**
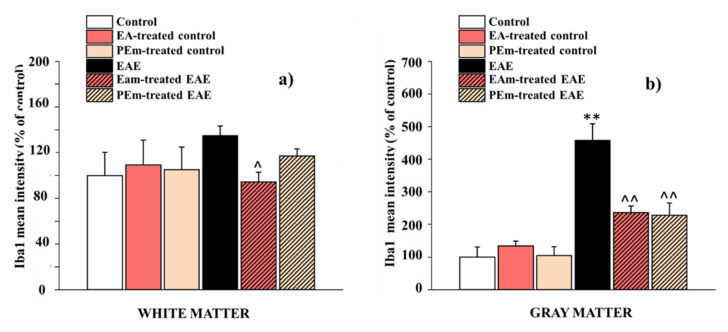
Effects of “in vivo” EAm and PEm treatments on the microglial profile in the gray and the white matters in the spinal cord. The histograms show the quantitative analysis for Iba-1 mean fluorescence intensity in the white matter (**a**) and in the gray matter (**b**) of the ventral spinal cord. Results are expressed as mean ± SEM of 4 animals per group and control is arbitrary taken as 100%. Note: ** *p* < 0.01 vs. control group; ^ *p* < 0.05 or ^^ *p* < 0.01 vs. EAE group.

In detail, we examined whether there were any differences on microglial profile between the gray and the white matter in the ventral spinal cord. For this purpose, four to five fields per animal, taken from the white or the gray matter, were analyzed for the mean fluorescence intensity. As shown in Figure 6a,b, a different pattern of action was displayed by the two treatments in order to counteract microglial activation. In fact, EAm showed its effectiveness in both white (94.50 ± 8.40% vs. 134.80 ± 8.70% in untreated EAE mice) and gray matter (236.30 ± 20.80 % vs. 457.40 ± 51.10 in untreated EAE mice), with a more pronounced effect on the latter (−29.90% in white matter vs. −48,34% in gray matter). On the other hand, PEm appeared to be effective only in the gray matter (228.30 ± 37.50 % vs. 457.40 ± 51.10 in untreated EAE mice; Figure 6b); however, a decrease, but not significant, in Iba-1 fluorescence intensity was also observed in the white matter (117.20 ± 6.00 % vs. 134.80 ± 8.70 % in untreated EAE mice; Figure 6a).

Regarding cell morphology, while in control groups, microglia exhibited a ramified morphology, showing more extensive branching and processes, EAE microglia became activated with cells that appear a little smaller than surveilling microglia. These cells showed an increased presence of short and thick branches on primary processes. No changes were observed in either group treated with pomegranate extracts (not shown).

**Figure 7:**
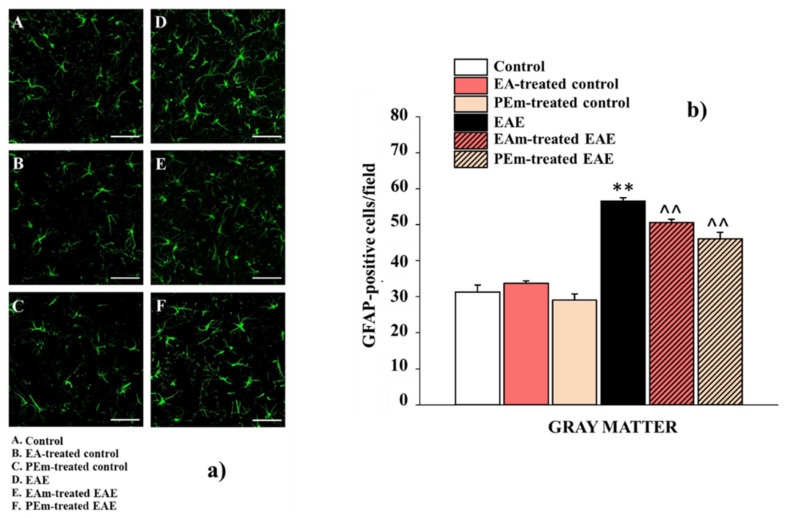
Effects of “in vivo” EAm and PEm treatments on the astrocytic profile in the spinal cord. (**a**) Representative images obtained by GFAP immunofluorescence histochemistry of the ventral spinal cord (lumbar portion; total magnification, 400×; scale bar, 50 µm). (**b**) Quantitative analysis for GFAP-positive cells/field in the ventral gray matter. Results are expressed as mean ± SEM of 4 animals per group. Note: ** *p* < 0.01 vs. control group; ^^ *p* < 0.01 vs. EAE group.

The activation of spinal astrocytes was evaluated by the expression of glial fibrillar acid protein (GFAP). EAE induced a significant increase in GFAP-positive cells (57 ± 1) compared to control animals (control, 31 ± 2; EAm-treated control, 34 ± 1; PEm-treated control, 29 ± 2). Treatment with EAm or PEm resulted in a numerical reduction of astrocytes, as demonstrated by immunohistochemistry (Figure 7a,b). In this case, PEm and EAm administration prevented the astrocytic response (EAm-treated mice, 51 ± 1; PEm-treated mice 46 ± 2). Next, the analysis for GFAP fluorescence intensity was conducted.

**Figure 8:**
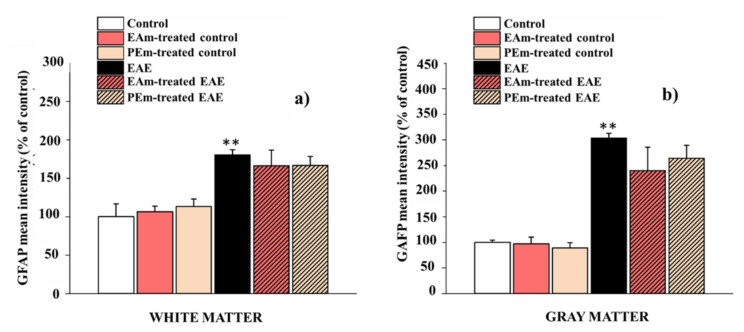
Effects of “in vivo” EAm and PEm treatments on “in vitro” astrocytic profile in the spinal-cord gray and white matter. The histograms show the quantitative analysis for GFAP mean fluorescence intensity in the white matter (**a**) and in the gray matter (**b**) of the ventral spinal cord. Results are expressed as mean ± SEM of 4 animals per group, and control is arbitrary, taken as 100%. Note: ** *p* < 0.01 vs. control group.

In this case, we observed a slight but not significant decrease of the stain intensity in all EAE groups (Figure 8a,b). These latter data can be explained due to the different morphology of the astrocytes: although they showed their characteristic star shape in all groups, an increasing number of their cytoplasmic processes was observed in EAE groups.

**Figure 9:**
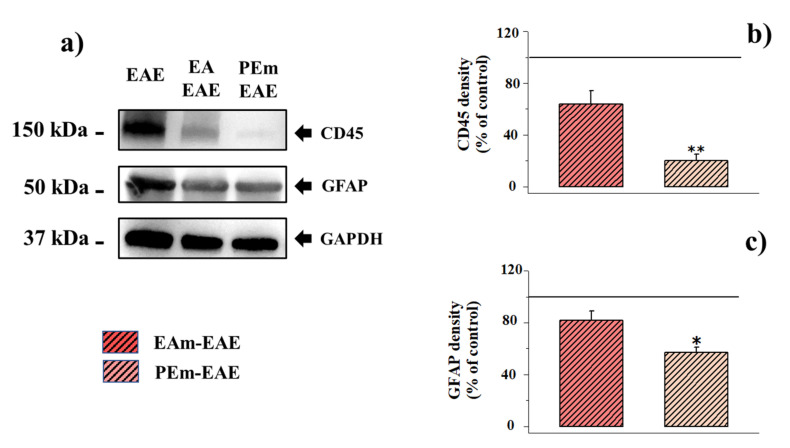
Effect of “in vivo” EAm and PEm treatments on the content of CD45 and of the glial fibrillar astrocytic protein (GFAP) in EAE mouse spinal-cord homogenates. (**a**) Representative Western blot of the immunostainings for CD45, GFAP and glyceraldehyde-3-phosphate dehydrogenase (GAPDH) in untreated, EAm-treated and PEm-treated EAE mouse spinal-cord homogenates. The protein GAPDH was used as the internal control. The blot is representative of the analysis on lysates from 6 animals for each experimental group. (**b**) Quantification of the change of CD45 density in the spinal-cord homogenates of EAm-treated (*n* = 6 mice) and PEm-treated (*n* = 6 mice) EAE mice versus the untreated ones (*n* = 6). Results are calculated as CD45 ÷ GAPDH ratio and are expressed as percentage of the respective ratio in untreated EAE mice (1.12 ± 0.22). Data are expressed as mean ± SEM. (**c**). Quantification of the change of GFAP density in the spinal cord homogenates of EAm-treated (*n* = 6 mice) and PEm-treated (*n* = 6 mice) EAE mice versus that of untreated ones (*n* = 6). Results are calculated as GFAP ÷ GAPDH ratio and are expressed as percentage of the respective ratio in untreated EAE mice (3.52 ± 0.41). Data are expressed as mean ± SEM. Note: * *p* < 0.05 versus untreated EAE mice; ** *p* < 0.01 versus untreated EAE mice. EA and PEm administration did not cause significant changes in the expression of the CD45 and the GFAP protein densities in control mice.

To confirm the impact of EAm and PEm administration on astrocytosis and lymphocytes infiltration, we analyzed the density of the GFAP and of CD45 (here used as a marker of lymphocytes) proteins in the spinal cord lysates of both untreated and treated EAE mice. The glyceraldehyde-3-phosphate dehydrogenase (GAPDH) protein was used as an internal standard to quantify the changes in GFAP and CD45 densities in the lysates from the different animals. The results show that the CD45 density in the spinal-cord lysate in PEm-treated EAE mice was hugely reduced when compared to untreated EA mice and slightly, although not significantly, affected in EAm-EAE (Figure 9a,b). Similarly, the GFAP immunopositivity was significantly hampered in PEm-treated EAE mice, but not in EAm-administered ones (Figure 9a,c).

EAm and PEm treatment did not cause significant changes in the protein density in the non-immunized control mice (Table 4).

## 3. Discussion

Pomegranate peels, obtained from the processing industry of pomegranate, represent an expensive disposal problem but also a promising source of nutraceuticals, particularly ETs, to be exploited for human health and well-being. In this work, compared to the previous one from Reference [18], the whole phytocomplex extracted by green technology from pomegranate peels was formulated and tested, with the aim to valorize this agro-industrial waste in a view of sustainability and circular economy. To improve the long-term stability of the aqueous peel extract (PE) obtained by PUAE, a spray-drying microdispersion, using pectin as polymeric matrix, namely another example of compound recovered from an agro-industrial waste, was realized. The formulated extract (PEm) was proven to be stable and maintained its antiradical activity for a period of 6 months of storage at room temperature and in the dark. PEm was titrated in punicalagin as a marker compound of the total content of ellagitannins of the phytocomplex, showing a content higher of 100 μg/mL. Furthermore, the HPLC sample profiling (ESI/MS–MS/MS) allowed us to identify thirty-four bioactive compounds, mainly ETs and flavonols.

PEm was found to control the clinical symptoms and some of the pathological hallmarks of EAE. In PEm-treated EAE mice, the gravity of the clinical signs did not get worse during the progression of the disorder; rather, at the acute stage of disease (23 d.p.i.), they were significantly lower than those detected in untreated EAE animals.

The reduction of the gravity of the clinical symptoms is, in general, predictive of an amelioration of the central histopathological derangements, as indeed reported in recent studies which demonstrated a clear recovery of myelin production in EAE mice that were administered with nutraceutics containing EA and pomegranate products [34,35,36]. Different from those studies, however, we did not observe an overt restoration of central demyelination or a significant reduction of the inflammatory infiltrates in the spinal cord of PEm-treated EAE mice, but rather a clear diminution of pathological hallmarks pivotal to the progression of these signs. We are referring to the spinal microgliosis and astrocytosis, which are significantly reduced in the PEm-treated EAE mice, particularly into the grey matter, as well as to the infiltrating lymphocytes that are recruited from the periphery to the CNS and act in concert with resident glial cells and astrocytes to dictate the progression of EAE. These cells enter the CNS because of the permeabilization of the blood–brain barrier to sustain local inflammation and degenerative processes. The infiltration of lymphocytes in the CNS, highlighted as an increased density of the CD45 protein, was found to be largely diffuse in the spinal cord of EAE mice, as indeed expected, but significantly decreased in PEm-treated EAE mice. Similarly, a clear reduction of the activation of astrocytes and glial cells (quantified as changes in the density of Iba1 and GFAP stainings in the spinal cord of EAE mice, respectively) emerged in the PEm-treated EAE mice, again predictive of a slowing-down of the disease progression [40,41,42]. We propose that the amelioration of these signs provides a cellular rationale to support the use of the PEm formulation as dietary supplementation during MS. As already introduced, our observations differ by a quantitative point of view from other recent results in the literature [30,31,32]. Differences in the formulation, in the posology (in those works, the nutraceutics were administered with a prophylactic protocol, starting from mice immunization) and in the experimental approach might account for the discrepancy: future studies will be dedicated to evaluating these aspects.

Notably, the EAm-induced effects were largely comparable to those caused by PEm, with the exception of GFAP and CD45 staining, for which the EAm was less efficacious, allowing us to speculate that the health effects could be ascribed to EA which is present in both the formulations and is proposed to mediate most of the cellular/molecular effects underlying the beneficial effects of pomegranates [10,30,31,32]. Although attractive, the hypothesis deserves some caution, particularly when considering the low bioavailability of EA, which is rapidly metabolized to urolithins that have a more favorable pharmacokinetic profile and could have systemic effects. Please note, the rapid metabolization of EA and ETs also raises the question of whether the two formulations could exert a central effect or, rather, if their protective activities (or those elicited by the main metabolites) preferentially occur peripherally. The point is intriguing, particularly when considering recent findings showing that EA and its main metabolites control the composition of the gut microbiota, influencing positively central pathological signals, particularly demyelination, but also reducing the lymphocyte infiltrates [8]. In this regard the clear reduction of the CD45 staining detected in the spinal cord of PEm and EAm-treated EAE mice might be consistent with peripheral events, that develop at peripheric lymphatic tissues, including the gut and the spleen, and reverberate positively in the CNS. Again, further studies are required to address this issue.

Another important result of the present study is that the health-promoting effects of PEm and EAm are observed with a therapeutic regimen (i.e., by administering both the formulations from the early clinical signs, at 10 ± 1 d.p.i., till the chronic stage of disease). The therapeutic regimen could be potentially less efficacious than a prophylactic one [8,30], since it should not prevent, but, rather, control the progression of the disorder. Nonetheless, the treatment is more consistent with the drug regimen/nutraceutic supplementation that is usually adopted by MS patients, who approach therapies and even dietary integrations once precocious clinical symptoms become evident and a diagnosis is proposed. The therapeutic protocol represents, therefore, an appropriate approach to study the efficacy of a food-integration for the cure of the MS.

## 4. Materials and Methods

### 4.1. Pomegranate Peels

Pomegranate fruits (Wonderful certified cultivar) were collected from Masseria Frutti Rossi (https://lomesuperfruit.com/it/home, 20 November 2021), one of the major Italian pomegranate producers (Castellaneta Taranto, Italy). Fruits were collected at full maturity (Maturity Index, MI= 14.5 ± 2.5) and immediately processed: the corresponding peels were manually separated during the juice production as waste materials. Peels were dried for 48 h at 40 °C in a traditional heating oven (Binder FED53, Goettingen, Germany) (Residual moisture: < 10%) and then finely ground in a mill by a Grindomix 200 M (Retsch, Haan, Germany) for 20 s at 5000 rpm. The distribution of peels particle size was determined by a 150 μm sieve system to obtain a fine and homogeneous powder.

### 4.2. Preparation of the Aqueous Peel Extract by Pulsed Ultrasound-Assisted Extraction (PUAE)

A sonicator Hielscher UP200St (Teltow, 171 Germany) with maximum nominal output power of 200 W, frequency of 26 kHz, and with a titanium probe (7 mm) was used for the ultrasound-assisted extraction of ellagitannins from pomegranate peels. The extract was obtained by ultrasonication in pulse modality (PUAE) at optimized conditions previously described by the Authors with slight modifications [35]. Briefly, only water was used as extraction solvent; time of extraction, 10 min; solvent/peels ratio, 40/1 mL/g; amplitude, 50 W; duty cycle, 80% (pulse duration/pulse interval ratio 4/1); temperature, <50 °C (using an ice bath). In PUAE, the ultrasound processor worked intermittently during the extraction process (active time vs. inactive time), allowing us to reduce the operating temperature, to preserve thermolabile compounds and to limit the occurrence of alterations [43]. The obtained extract (PE) was centrifuged at 5000 rpm for 20 min, filtered by Buchner (filter paper Whatman n. 1) and stored frozen (−20 °C) until the analysis time. High-purity water (HPW) produced with a Millipore Milli-Q system was used throughout. Solvents and standards used for chromatographic purposes were of HPLC grade and were purchased from Sigma-Aldrich (Milwaukee, Brookfield, WI, USA). Ellagic acid and punicalagin were purchased from Sigma-Aldrich (Germany) and were used without further purification. Low methoxylated pectin (LMP with a DE < 50%) derived from citrus peels was purchased from Alfa Aesar (Germany).

### 4.3. Formulation of the Aqueous Peel Extract by Spray-Drying Technology

Aqueous pomegranate-peel extract (PE) obtained by PUAE was formulated to improve its long-term stability, using low methoxylated (LM) pectin (degree of esterification < 50%) by spray-drying. A total of 200 mL of PE was heated (40 °C) for 10 min before adding 0.9 g of LM pectin and then maintained under continuous mixing at room temperature for 30 min in order to homogenate the dispersion. The obtained homogenated dispersion was spray-dried in a laboratory-scale BUCHI Mini Spray Dryer B-290 (Flawil, Switzerland) with a nozzle atomizer and a chamber length and diameter of 60 cm and 16.5 cm, respectively. According to optimum experimental conditions developed from the authors in a previous study [18], PEm was obtained by using inlet air temperature equal to 160 °C, flow rate of drying air equal to 600 L/h and feed rate of 11 mL/min. The microencapsulation efficiency (EE %) of the formulation process was calculated by comparing the punicalagin content of the aqueous peels extract solid microdispersion (PEm) with the punicalagin content of the corresponding aqueous peels extract (PE).

### 4.4. Preliminary Characterization of the Aqueous Peel Extract Solid Microdispersion (PEm)

PEm was characterized by several analytical determinations. Moisture was gravimetrically determined by a MA40 thermo-balance (Sartorius, Germany). Bulk density was determined by accurately weighing 2 g of powder in a 50 mL graduated cylinder and dividing the mass of the powder by the volume [35]. Solubility was determined by adding increasing amounts of powder to 1 mL of Milli-Q water thermostated at 25 °C and agitating by vortex until complete solubilization. The powder was considered soluble when the time of solubilization was not longer than 5 min. Radical scavenging activity (RSA) was determined by the in vitro DPPH· (1,1-diphenyl-2-picryl-hydrazyl) assay [22]. Briefly, 250 μL of each extract was transferred into a 10 mL volumetric flask and was mixed with a daily prepared 10^−4^ M DPPH solution (in methanol). After 30 min of incubation in the dark and at room temperature, the residual absorbance was read at 515 nm against a blank (solution without radical). The initial DPPH concentration was measured by using control samples obtained by diluting 250 μL of methanol with the DPPH solution in a 10 mL volumetric flask. The absorbance measurements were transformed in antioxidant activity by using ascorbic acid as reference standard. A five-point calibration curve in ascorbic acid (*R*^2^ = 0.9975) was obtained. RSA was expressed as ascorbic acid equivalent antioxidant capacity (AEAC: mg ascorbic acid/g of powder). Two replicated determinations were performed, and results were reported as mean ± standard deviation (SD).

### 4.5. Chromatographic Characterization of the Pomegranate Phyto-Complex (PE and PEm): HPLC–DAD Analysis

HPLC analysis were carried out with an HPLC Agilent 1100 Liquid Chromatograph equipped with a Diode Array Detector (LC–DAD, Agilent Technologies, Paolo Alto, Santa Clara, CA, USA). A C-18 Kromasil 100^®^ (Akzo-Nobel, Amsterdam, NL) column (250 × 4.6 mm, particle size 5 μm) was employed. The determination and quantification of both EA and Punicalagin was realized, as reported by Millo et al. [44], for punicalagin detection, with slight modifications. The mobile phase was composed by solvent A, 0.1% fosforic acid in water; and solvent B, methanol, using 1 mL/min of flow rate, 25 °C of column temperature. The linear gradient was 0 to 27% for solvent B for 30 min, 27 to 55% solvent B for the next 15 min, 55 to 100% solvent B for 5 min, maintained 100% solvent B for 10 min, and followed by a 10 min re-equilibration time back to 0% solvent B. Then 20 μL of each sample, previously filtered by an RC membrane syringe filter (0.45 μm), was injected into the column per cycle. Punicalagin was detected and quantified, as sum of punicalagin a and b, at 254 nm (retention time = 18 and 22 min, respectively) by external standard quantification with a four-point regression line (y = 0.02x − 0.4744; R^2^ = 0.9999), in the range between 5 and 100 μg/mL. EA was detected and quantified at 254 nm (retention time= 45 min) by external standard quantification with a four-point ellagic acid regression line (y= 250.3x − 64.4; R^2^= 0.9998), in the range between 1 and 50 μg/mL. Results were expressed as µg of punicalagin and ellagic acid in mL of pomegranate extract.

### 4.6. Chromatographic Characterization of the Pomegranate Phyto-Complex (PE and PEm): HPLC–MS Analysis

High-performance liquid chromatography coupled with mass spectrometry (HPLC–MS and MS/MS) was performed by using an Agilent 1100 HPLC-MSD Ion Trap XCT system, equipped with an electrospray ion source (HPLC-ESI-MS) (Agilent Technologies, Santa Clara, CA, USA). Separation of extracts was performed on a Zorbax SB C-18 column 1 mm × 150 mm with 3.5 μm particle size (Agilent Technologies). As eluents, water (eluent A) and acetonitrile (eluent B), both with added 0.1% formic acid, were used. The gradient employed was initial, 2% B; 0–5 min, linear up to 10% B; 15–15 min, isocratic 10% B, 10–15 min, linear up to 20% B; 20–25 min, liner up to 30% B; 25–40 min, linear up to 60% B; 40–45 min, linear up to 100% B; and 45–50 min, isocratic 100%. The column was then reconditioned at initial condition in 15 min. The flow rate was set to 30 µL/min with a column temperature of 30 °C. The injection volume was 8 μL. Ions were detected in the negative ion mode, in the 200–1600 m/z range. Ion charged control with a target ion value of 50,000 and an accumulation time of 300 ms were set. A capillary voltage of 3500 V, nebulizer pressure of 10 psi, drying gas of 8 L/min, dry temperature of 325 °C, and 3 rolling averages (averages: 5) were the parameters set for the MS detection. MS/MS analysis was conducted by using an amplitude optimized time by time for each compound.

### 4.7. Animals and EAE Induction

Mice (female, strain C57BL/6J, seven weeks old) were obtained from Charles River (Calco, Italy) and housed at the animal facility of the Department of Pharmacy, Section of Pharmacology and Toxicology, School of Medical and Pharmaceutical Sciences, University of Genoa (authorization n. 484 of 2004, June 8th). EAE was induced in mice as previously described [45]. Briefly, mice were injected subcutaneously with incomplete Freund’s adjuvant containing 200 μg myelin oligodendrocyte protein 35–55 (MOG_35–55_; Espikem S.r.l, Prato, Italy) peptide and 8 mg/ml Mycobacterium tuberculosis (strain H37Ra; Becton, Dickinson and Company, Franklin Lakes, NJ, USA), followed by intraperitoneal administration of 400 ng of Pertussis toxin (List Biological Laboratories, Campbell, CA, USA) on day 0 and after 48 h. Non-immunized control mice underwent the same procedure in the absence of MOG_35–55_. Clinical scores (0, healthy; 1, limp tail; 2, ataxia and/or paresis of hindlimbs; 3, paralysis of hindlimbs and/or paresis of forelimbs; 4, tetra-paralysis; and 5, moribund or death) were monitored, concomitantly, to the control of the weight and of the daily amount of drinking water/solution. Mice were sacrificed at 23 days post-immunization (d.p.i) by cervical dislocation, and the spinal cords were rapidly removed for in vitro studies. The animal procedures were approved by the Italian Ministry of Health (authorization n° 503/2021-PR) in accordance with the European legislation (CEE, September 22, 2010, no. 2010/63/EU), with the Italian legislation (L.D. no. 26, 4 March 2014 and 116/1992) and the 3Rs rules. All efforts were made to minimize animal suffering and to use the minimal number of animals necessary to produce reliable results.

### 4.8. Animal Treatment

Control and EAE mice were randomly assigned the following experimental groups: untreated mice, EA-treated mice and PEm-treated mice. EA (50 mg/kg/day) and PEm (containing EA to deliver daily 50 mg/kg ellagic acid) were given orally, dissolved in the drinking water for 14 days, starting from 10 to 23 d.p.i. Animals were controlled for the drinking-solution intake and the body weight during the treatment.

### 4.9. Western Blot Analysis

Mouse spinal cord homogenates were prepared as previously described [18] and then separated by SDS–10% PAGE (40 µg/lane) and blotted onto PVDF membrane. Membranes were blocked for 1 h at room temperature in Tris-buffered saline-Tween (t-TBS: 20 mM Tris, pH 7.4, 150 mM NaCl and 0.05% Tween 20), containing 5% (w/v) non-fat dried milk, and then probed with the following primary antibodies overnight at 4 °C: rabbit anti-CD45 (1:2000, Cell Signaling Technology, Danvers, MA, USA), mouse anti-GFAP (1:5000, Sigma-Aldrich, St. Louis, MO, USA) and rabbit anti-GAPDH (1:5000, Cell Signaling Technology, USA). After washes in t-TBS, membranes were incubated for 1 h at room temperature with the proper horseradish peroxidase-linked secondary antibodies (1:20000). Immunoblots were visualized by using an ECL (enhanced chemiluminescence) Western blotting detection system. Images were acquired by using the Alliance LD6 images capture system (Uvitec, Cambridge, UK) and analyzed with UVI-1D software (Uvitec, Cambridge, UK).

### 4.10. Histological Analysis

For histological evaluation, formalin post-fixed lumbar spinal cord segments were processed for paraffin embedding. After rehydration, 10 µm–thick sections were stained with Luxol Fast Blue (LFB) and with Hematoxylin/Eosin (H&E), as previously described [44].

LFB staining was performed in order to evaluate the density of the spinal-cord ventral white matter. The area stained by LFB was quantified by using ImageJ software (ImageJ, National Institute of Health, USA, https://imagej.nih.gov/ij/, accessed on 20 November 2021) and expressed as relative myelination area.

H&E staining was performed for the evaluation of the inflammatory infiltration, according to the score chart from 0 (no inflammation) to 4 points (severe inflammation), as reported by Liu and colleagues in 1998 [46].

Furthermore, Giemsa staining was implemented for the identification of the mast cells. Briefly, paraffin-embedded sections were dewaxed in xylene and hydrated to distilled water. After rinsing in methanol for 2 min, sections were immersed in preheat working Geimsa solution (14% Geimsa stock solution (Bio-Optica, Milan, Italy), 76% distilled water and 10% methanol) for 2 min. After dipping in 1% acetic acid, slides were quickly washed in distilled water and rinsed three times in methanol. Finally, sections were cleared in xylene and mounted.

For all histopathological setups, five different sections of each animal were analyzed under light microscopy by Olympus BX40 microscope (Olympus, Milan, Italy), and 40X fields per section were captured by using a digital camera Olympus DP50 (Olympus, Milan, Italy).

### 4.11. Immunohistochemistry

To perform immunofluorescence staining for spinal astrocyte and microglia analysis, formalin post-fixed lumbar spinal cord segments were cryoprotected in 30% sucrose solution at 4 °C and cut transversally into 7 µm–thick slabs. After incubation for 1 h in blocking solution (Bio-Optica, Italy), slide-mounted cryostat sections were incubated with rabbit anti-GFAP (1:200; Dako, Dako, Glostrup, Denmark) or rabbit anti-Iba1 (1:200; Wako Chemicals, Richmond, VA, USA) o/n at 4 °C. The following day, after rinsing in PBST, tissue sections were incubated with the appropriate secondary fluorescent antibody, Alexa Fluor488- or 568-conjugated goat anti-rabbit IgG (1:500; Invitrogen, Italy), for 1 h, at room temperature, and with DAPI (1:2000; Invitrogen, Italy) to visualize cell nuclei. Specificity of each assay was tested by omitting the primary antibody.

Sections were examined under Leica DM6000B fluorescence microscope, and images were captured with a DFC350FX camera.

Quantitative analysis, using ImageJ software (ImageJ, National Institute of Health, Bethesda, MD, USA, https://imagej.nih.gov/, accessed on 20 November 2021), was performed on each sample by collecting at least three independent fields through a 20 × 0.5 NA objective. For each field, equally sized (296.24 × 134.92 μm^2^) sub-areas taken either from the white matter or the gray matter were selected and separately analyzed. The results were expressed both as mean fluorescence intensity and cell number per field. DAPI counterstaining of cell nuclei was not reported in the illustrative images. 

### 4.12. Calculation and Statistical Analysis

The univariate statistical analysis was carried out by using the Sigma Plot program. Analysis of variance was performed by ANOVA followed by Dunnett’s or Tukey’s multiple comparison tests as appropriate. Direct comparison was performed with the student’s *t*-test. Data were considered significant for *p* < 0.05 at least.

## 5. Conclusions

Our study proposes a new formulation of pomegranate peels that valorize an agro-industrial waste in a view of sustainability and circular economy, providing “in vivo” and “in vitro” data supporting its nutraceutical properties to be exploited to improving human health and well-being in MS patients. Our results prove the efficacy of PEm and EAm in reducing the progression of the EAE in mice, indicating that its therapeutic daily supplementation positively reverberate on “peripheral to CNS” signalings, reinforcing the resilience to disease progression. Overall, our study provides proof of concept to propose the translation to clinic of the PEm as a botanical dietary supplementation for disease-modifying drug treatments in MS patients.

## Figures and Tables

**Table 1:** Preliminary characterization of new pomegranate-peel extract (namely PEm).

Determination	PEm
*Moisture (%)*	13.67 ± 0.01
*Solubility (mg/mL)* *Bulk density*	50.00 ± 0.10 0.20 ± 0.05
*Radical scavenging activity (RSA: mg AEAC/g powder)*	537.43 ± 10.78

Data are expressed as mean ± SD; AEAC, ascorbic acid equivalent antioxidant capacity.

**Table 2:** Ellagic acid (EA) and punicalagin contents of the aqueous peel extract (PE) and the corresponding solid microdispersion (PEm).

SAMPLE	PUNICALAGIN	ELLAGIC ACID
	μg/mL	
**PE**	692.2 ± 19.4	57.8 ± 2.9
**PEM**	103.4 ± 8.2	2.8 ± 0.3
	* EE %= 14.9	

* The microencapsulation efficiency (EE%) of the formulation process, calculated by comparing the punicalagin content of PEm with the punicalagin content of the corresponding PE, was reported too.

**Table 3:** Compounds identified by ESI/MS–MS/MS analysis. For each molecule, the HPLC retention time (RT), the m/z ratio in the negative ion mode ([M-H]-) and the fragmentation pattern are reported. The fragments are reported for their relative abundance in descending order.

Peak	RT (min)	[M-H]^−^ m/z	MS/MS Fragments	Tentatively Identified Compound
1	5.3	649	301,497	Galloyl-HHDP-gluconate (lagerstannin C) isomer [30]
2	5.4	481	301,275	HHDP_hexoside [30,31]
3	5.9	481	301,275	HHDP_hexoside [30,31]
4	7.5	481	301,275	HHDP_hexoside [30,31]
5	8.8	783	601,721,765,481,301	bis-HHDP-hexoside (Pedunculagin I isomer) [29,30]
6	8.9	649	301, 497	Galloyl-HHDP-gluconate (lagerstannin C) isomer [30]
7	9.7	735	675, 657, 603, 543, 381	Unknown
8	9.9	645	585, 567, 513, 453, 301	Unknown
9	10.8	633	301, 481, 275, 249	Galloyl-HHDP-hexoside isomers [29,30]
10	11.5	781	601.721	Punicalin isomer [30]
11	11.6	781	601.721	Punicalin isomer [30]
12	12	783	601,721,765,481,301	bis-HHDP-hexoside (Pedunculagin I isomer) [29,30]
13	12.2	649	301,497	Galloyl-HHDP-gluconate (lagerstannin C) isomers [30]
14	12.5	633	301,481,275,249	Galloyl-HHDP-hexoside isomers [29,30]
15	12.7	1083	601,781,721	Punicalagin isomer [30]
16	13.3	783	301,481,765	Pedunculagin I isomer [29,30]
17	14.4	951	907,783, 765,481	HHDP-valoneoyl-glucoside [31]
18	15.2	1083	601, 781, 721	Punicalagin α [30]
19	16.7	783	481, 301	bis-HHDP-hexoside (Pedunculagin I isomer) [30]
20	17.5	633	301, 481, 275, 249	Galloyl-HHDP-hexoside isomers [29,30]
21	18.3	783	481, 301	bis-HHDP-hexoside (Pedunculagin I isomer) [30]
22	19.0	785	301, 633, 483, 615	Digalloyl-HHDP-hexoside (pedunculagin II) [29,31]
23	19.6	1083	601, 781, 721	Punicalagin β [30]
24	23.2	801	649, 499, 349, 301	Digalloyl-HHDP-glucoside (punigluconinisomer) [29]
25	23.8	965	933	Castalagin derivate [29]
26	24.5	801	649, 499, 349, 301	Digalloyl-HHDP-glucoside (punigluconinisomer) [29]
27	26.3	785	483, 633, 301, 615	Digalloyl-HHDP-hexoside (pedunculagin II) isomer [29]
28	27.1	633	301, 463, 275, 245	Corilagin [31]
29	28.5	463	301	Ellagic acid-glucoside [29]
30	29.4	614	463	Unknown
31	30.2	447	301	Ellagic acid-deoxyhexoside [29]
32	30.6	433	301	Ellagic acid-pentoside [29]
33	31.0	593	285	Kaempferol rutinoside [29]
34	31.5	609	301	Rutin [31]

**Table 4:** Effect of “in vivo” EAm and PEm treatments on the CD45 and of GFAP protein density in spinal-cord homogenates of control mice.

	CD45/GAPDH Ratio	% of Change	GFAP/GAPDH Ratio	% of Change
Control	0.083 ± 0.017	--	4.04 ± 0.60	--
EA-treated control	0.065 ± 0.023	−22.87%	3.43 ± 0.22	−14.92%
PEm-treated control	0.085 ± 0.022	+2.23%	4.20 ± 0.53	+4.10%

Results are calculated as CD45 ÷ GAPDH ratio and GFAP ÷ GAPDH ratio in both treated and untreated control mice and are also expressed as percentage of the respective ratio in untreated control mice. Data are expressed as mean ± SEM.

## Data Availability

The data presented in this study are available in the article.

## Sample Availability

Samples of the compounds are not available from the authors.

## Abbreviations

MS	multiple sclerosis
EA	ellagic acid
ETs	ellagitannins
PE	pomegranate extract
PEm	pomegranate extract solid microdispersion
EAm	EA solid microdispersion
EAE	experimental autoimmune encephalomyelitis
PUAE	Pulsed Ultrasound-Assisted Extraction
LM	low methoxylated
d.p.i.	days post immunization
CNS	central nervous system
LFB	Luxol Fast Blue
H&E	Hematoxylin and Eosin
Iba1	Ionized Calcium Binding Adaptor 1
GFAP	glial fibrillar acid protein
RSA	radical scavenging activity
