# Correction: Vallarino et al. Healthy Properties of a New Formulation of Pomegranate-Peel Extract in Mice Suffering from Experimental Autoimmune Encephalomyelitis. *Molecules* 2022, *27*, 914

**DOI:** 10.3390/molecules30081769

**Published:** 2025-04-15

**Authors:** Giulia Vallarino, Annalisa Salis, Elena Lucarini, Federica Turrini, Guendalina Olivero, Alessandra Roggeri, Gianluca Damonte, Raffaella Boggia, Lorenzo Di Cesare Mannelli, Carla Ghelardini, Anna Pittaluga

**Affiliations:** 1 Department of Pharmacy, University of Genoa, Viale Cembrano, 4 I, 16148 Genoa, Italy; vallarino@difar.unige.it (G.V.); turrini@difar.unige.it (F.T.); olivero@difar.unige.it (G.O.); roggeri@difar.unige.it (A.R.); pittalug@difar.unige.it (A.P.); 2 Department of Experimental Medicine, Section of Biochemistry, University of Genova, Viale Benedetto XV 1, 16132 Genoa, Italy; annalisa.salis@unige.it (A.S.); Gianluca.Damonte@unige.it (G.D.); 3 Department of Neuroscience, Psychology, Drug Research and Child Health, Neurofarba, Pharmacology and Toxicology Section, University of Florence, Viale Pieraccini 6, 50139 Florence, Italy; elena.lucarini@unifi.it (E.L.); lorenzo.mannelli@unifi.it (L.D.C.M.); carla.ghelardini@unifi.it (C.G.); 4 Center of Excellence for Biomedical Research (CEBR), University of Genova, Viale Benedetto XV 9, 16132 Genoa, Italy

**Error in Figure**

In the original publication [1], there was a mistake in Figure 5. During the preparation of the figure, images obtained using the same specimen (from “Control group”) were unintentionally used as representative pictures for both groups: the “Control” (panel A) and the “PEm-treated control” (panel C). For this reason, the panels show a common area. The corrected Figure 5 appears below. The authors state that the scientific outcome is unaffected, as the conclusions drawn from the originally published figure are consistent with those drawn from the correct one. In both representations, it emerges that the PEm treatment does not modify the number of Iba1-positive cells in the control conditions. 

This correction was approved by the Academic Editor. The original publication has also been updated.

## Figures and Tables

**Figure 5:**
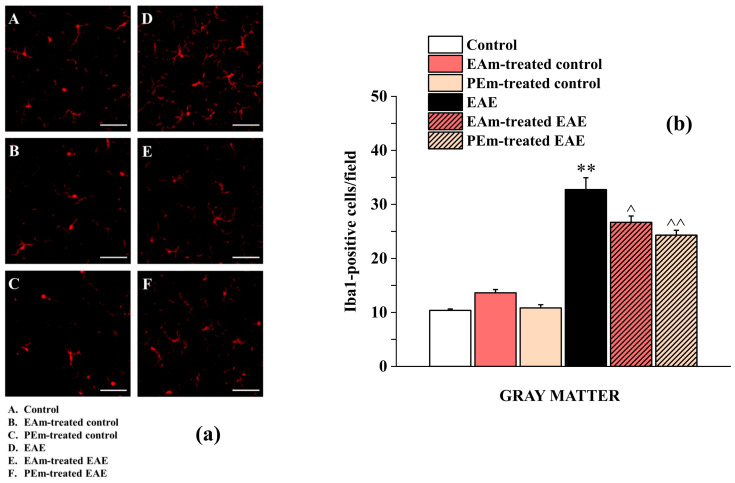
Effects of “in vivo” EAm and PEm treatments on the microglial profile in the spinal cord. (**a**) Representative images obtained by Iba-1 immunofluorescence histochemistry of the ventral spinal cord, lumbar portion (total magnification, 400×; scale bar, 50 µm). (**b**) Quantitative analysis for Iba-1-positive cells/field in the ventral gray matter. Results are expressed as mean ± SEM of *n* = 4 animals per group. Note: ** *p* < 0.01 vs. control group; ^ *p* < 0.05 or ^^ *p* < 0.01 vs. EAE group.
